# A novel reporter system for neutralizing and enhancing antibody assay against dengue virus

**DOI:** 10.1186/1471-2180-14-44

**Published:** 2014-02-18

**Authors:** Ke-Yu Song, Hui Zhao, Zhen-You Jiang, Xiao-Feng Li, Yong-Qiang Deng, Tao Jiang, Shun-Ya Zhu, Pei-Yong Shi, Bo Zhang, Fu-Chun Zhang, E-De Qin, Cheng-Feng Qin

**Affiliations:** 1 Department of Virology, State Key Laboratory of Pathogen and Biosecurity, Beijing Institute of Microbiology and Epidemiology, Beijing 100071, China; 2 Department of Microbiology and Immunology, School of Medicine Jinan University, Guangzhou 510632, China; 3 Novartis Institute for Tropical Diseases, Singapore 138670, Singapore; 4 State Key Laboratory of Virology, Wuhan Institute of Virology, CAS, Wuhan 430072, China; 5 Guangzhou No.8 People’s Hospital, Guangzhou 510060, China

**Keywords:** Dengue virus, Neutralizing antibody, Enhancing antibody, Luciferase assay

## Abstract

**Background:**

Dengue virus (DENV) still poses a global public health threat, and no vaccine or antiviral therapy is currently available. Antibody plays distinct roles in controlling DENV infections. Neutralizing antibody is protective against DENV infection, whereas sub-neutralizing concentration of antibody can increase DENV infection, termed antibody-dependent enhancement (ADE). Plaque-based assay represents the most widely accepted method measuring neutralizing or enhancing antibodies.

**Results:**

In this study, a novel reporter virus-based system was developed for measuring neutralization and ADE activity. A stable *Renilla* luciferase reporter DENV (Luc-DENV) that can produce robust luciferase signals in BHK-21 and K562 cells were used to establish the assay and validated against traditional plaque-based assay. Luciferase value analysis using various known DENV-specific monoclonal antibodies showed good repeatability and a well linear correlation with conventional plaque-based assays. The newly developed assay was finally validated with clinical samples from infected animals and individuals.

**Conclusions:**

This reporter virus-based assay for neutralizing and enhancing antibody evaluation is rapid, lower cost, and high throughput, and will be helpful for laboratory detection and epidemiological investigation for DENV antibodies.

## Background

The four serotypes of dengue virus (DENV) belong to the genus *Flavivirus* within the family *Flaviviridae*[1]. The clinical manifestations of DENV infections cover a wide range of symptoms, from mild dengue fever (DF) to severe life threatening dengue hemorrhagic fever (DHF) and dengue shock syndrome (DSS) [2]. Commonly, DHF/DSS is associated with sequential DENV infection by different serotypes [3,4]. Annually, 50 to 100 million people in over 100 countries are infected with DENV and DHF/DSS can be fatal in up to 5% of affected individuals. No vaccine or specific antiviral drugs is currently available.

DENV is a typical positive-sense, single-stranded RNA virus. The genome is about 11 kb in length and encodes three structural proteins (C, prM and E) and seven non-structural proteins (NS1, NS2A, NS2B, NS3, NS4A, NS4B, and NS5). Neutralizing antibody is predominantly induced against E protein, and laboratory and clinical studies have demonstrated that protection of animals or individuals from DENV infection is best correlated to titer of neutralizing antibody (>1:10). However, pre-existing sub-neutralizing concentration of antibody or non-neutralizing antibody was also evidenced to enhance DENV infection in Fc gamma Receptor (FcγR) - positive cells and appears to be a risk factor for severe diseases. This phenomenon is known as antibody-dependent enhancement (ADE) infection [5,6]. Thus, human antibodies are believed to play distinct roles in controlling DENV infection. It is important to characterize antibody with neutralizing or enhancing activities against DENV for both basic and applied research.

Currently, plaque-based analysis is the most widely accepted method measuring neutralizing or enhancing antibodies [7] and has been recommended by the World Health Organization. However, this traditional method is time-consuming and labor intensive, and not suitable for large-scale samples analysis. Further, plaque-based assay can only be performed in cells that permit plaque forming and quantified by an operator-error prone manual readout based on the number of plaques. There is a great need of novel technology for characterizing DNEV neutralizing and enhancing antibodies in a simple, rapid, and high-throughput manner [8].

Recently, we have developed a stable luciferase reporter DENV (Luc-DENV) for antiviral high-throughput screening [9]. In this study, we aim to adapt the Luc-DENV for anti-DNEV neutralizing and enhancing antibodies evaluation. This newly developed reporter virus-based assay is validated using various known monoclonal antibodies (mAbs) and clinical samples from infected animal and patients, demonstrating well correlation with the traditional plaque-based assays.

## Results

### Development of Luc-based neutralizing assay

The Luc-DENV was developed by engineering the *Renilla* luciferase gene into the capsid-coding region by reverse genetic technology [9]. We have shown that Luc-DENV replicates efficiently in both mammalian and mosquito cells with high stability. As shown in Additional file 1: Figure S1 and Additional file 2: Figure S2, increasing amounts of luciferase signal were observed from 24 to 96 h post-infection in Luc-DENV infected BHK-21 and K562 cells.

**Figure 1:**
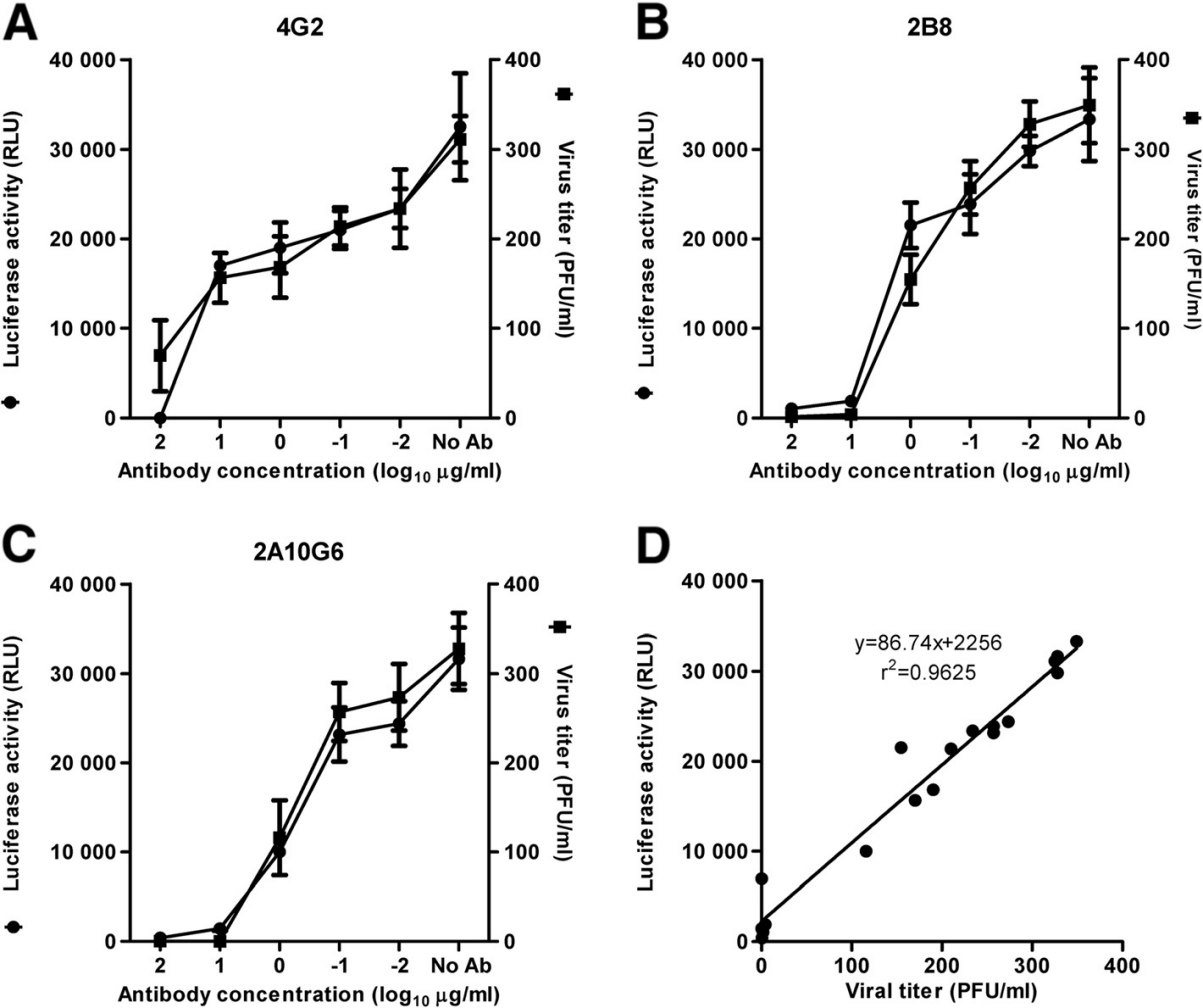
**Comparison of the new and conventional antibody neutralization assay system.** Neutralization activities mediated by various concentrations of mAbs (**A**: 4G2, **B**: 2B8, **C**: 2A10G6) specific for E protein of DENV in BHK-21 cells were performed with the new (square) and conventional (round) antibody neutralization assay system. Error bars indicate the standard deviations from two independent experiments. **(D)** Linear correlation between RLU and PFU values for neutralization assay.

To adapt Luc-DENV for neutralizing assay, we firstly assayed three identified neutralizing mAbs 4G2 [10], 2B8 [11] and 2A10G6 [11] by using plaque-based and Luc-based assay, respectively. Standard PRNT was performed in 12-well plates using 10-fold dilution of each mAb. The results showed that all three mAbs significantly reduced the numbers of plaques in a dose-dependent manner (Figure 1,ABC, right ordinate). The PRNT50 of 4G2, 2B8 and 2A10G6 was 8.55, 0.45 and 0.35 μg/mL, respectively. The RLU based assay was performed in the 12-well plate using the same dilutions of each mAb. The results demonstrated that all three mAbs significantly decreased RLU in a dose-dependent manner (Figure 1, ABC, left ordinate). LRNT50 of three mAbs calculated from a fitting curve were 6.80, 0.86 and 0.26 μg/mL, respectively, which was of the same order of magnitude with PRNT50. An unrelated mAb against EV71 showed no neutralization for both plaque and Luc-based assay (data not shown). Data fitting was made between values above. As expected, a linear correlation (R^2^ > 0.95) was demonstrated between PFU and RLU assay, and the linear equation between RLU and PFU is calculated as RLU = 86.74 PFU + 2256 (Figure 1D). Our results supported the application of Luc-based assay for neutralization antibodies against DENV.

### Development of Luc-based ADE assay

**Figure 2:**
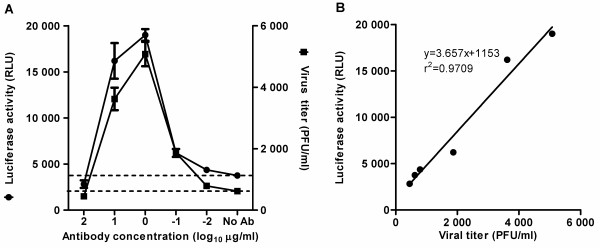
**Comparison of the new and conventional enhancing assay system. (A)** Enhancing assay of anti-E protein mAb 2A10G6 to DENV-2 in K562 cells with Luc-DENV. Luciferase activities (square) and PFU (round) were measured at 72 h after incubating virus–antibody complex with K562 cells. Error bars indicate the standard deviations from two independent experiments. **(B)** Linear correlation between RLU and PFU values in enhancing assay.

To develop the Luc-DENV for ADE assay, K562 cells were infected with Luc-DENV in the presence of serial 10-fold dilutions of 2A10G6. The viral titers in the supernatants were measured by standard plaque-based assay and Rlu-based assay, respectively. The results showed that the viral yield was markedly enhanced in the presence of 2A10G6 at dilutions ranging from 100 μg/mL to 0.01 μg/mL, and the peak enhancing was 8.19-fold at a concentration of 1.00 μg/mL (Figure 2A, right ordinate). The RLU assay showed similar pattern of enhancing, and the peak enhancing was 5.06-fold at a concentration of 1.00 μg/mL (Figure 2A, left ordinate), of the similar magnitude with plaque based assay. To get a linear equation between RLU and PFU, the results obtained with 2A10G6 were plotted on a scatter graph (Figure 2B). As expected, the enhancing antibody titer determined by RLU was linear correlated to PFU (R^2^ > 0.95), and the linear equation between RLU and PFU obtained was RLU = 3.657PFU + 1152, similar to the neutralizing equation. Together, these results indicated that this novel reporter system using Luc-DENV is readily for antibody neutralizing and enhancing assay with equivalent reliability to the conventional PFU-based assays.

### Validate the use of the assay with clinical samples

**Figure 3:**
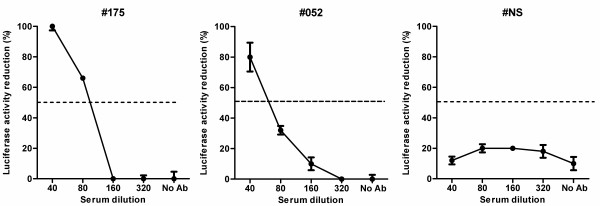
**Enhancing activity assay of monkey anti-DENV sera using the new assay system.** Samples #175 and #052 were obtained from subjects positive to DENV, and #NS (negative serum) was a sample from healthy subject as a negative control. Sera in various dilutions were mixed with Luc-DENV and incubated for 72 h. Luciferase activities were measured in lysed K562 cells to assay enhancing activities. Error bars indicate the standard deviations from two independent experiments.

**Figure 4:**
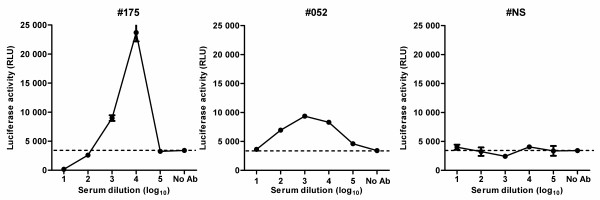
**Neutralization assay for monkey sera using the new assay system.** Samples #175 and #052 and #NS in various dilutions were mixed with 50 PFU Luc-DENV, after incubating for 24 h, mixtures were added to BHK-21 cells in 12-well plate. Luciferase activities were measured in lysed BHK-21 cells after 48 h incubating to assay neutralization activities. Error bars indicate the standard deviations from two independent experiments.

Finally, this RLU based assay was validated with clinical samples from immunized monkeys and patients. Neutralization assays were performed using 2-fold serial dilution sera in BHK-21 cells. For enhancing assay, sera were 10-fold serial dilution and assay was performed in K562 cells. Sera from Rhesus Monkeys (#175, #052) immunized with a live attenuated DENV-2 showed strong neutralizing activity, and LRNT50 was calculated to 100 and 70, respectively (Figure 3). Negative control (#NS) from healthy monkey showed no neutralizing activity as expected. Luc-based enhancement assay showed that both sera from immunized monkeys could significantly enhanced Luc-DENV replication at dilutions from 2 × 10^-2^ to 10^-5^ (#175), and 10^-1^ to 10^-5^ (#52), respectively. The enhancing activity of #175 is higher than that of #52. No enhancement was observed for #NS as expected (Figure 4).

**Figure 5:**
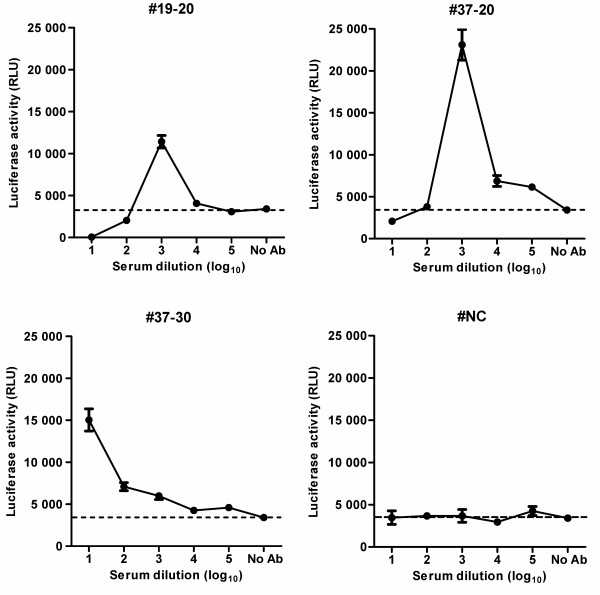
**Enhancing activity assay for patient sera using the new assay system.** Samples #19-20, #37-20 and #37-30 were obtained from Chinese subjects positive to DENV, with a sample from healthy people #NS as a negative control. Sera in various dilutions were mixed with Luc-DENV and incubated for 72 h, and luciferase activities were measured in lysed K562 cells to assay enhancing activities. Error bars indicate the standard deviations from two independent experiments.

Three convalescent sera from DF patients (#19-20, #37-20, #37-30) were validated with the newly developed assay in K562 cells. As shown in Figure 5, all three samples were able to enhance DENV infection at dilutions from 2 × 10^-2^ to10^-4^ (#19-20), 10^-2^ to10^-5^ (#37-20), and 10^-1^ to10^-4^ (#37-30), respectively. Negative control (#NC) from healthy adult in varying dilutions showed no impact on RLU as expected. Meanwhile, serum #19-20 and #37-20 showed strong neutralizing activities at a dilution of 10^-2^ or even lower, and LRNT50 was calculated to 80 and 10-fold dilution separately, whereas no neutralizing activity can be observed in serum #37-30 at detected dilutions. Together, these results indicate that the Luc-based assay is suitable for detecting both neutralization and ADE activity of immune sera from vaccinated or infected individuals.

## Discussion

A reliable, rapid, and high-throughput assay for DENV neutralization antibodies is critical for laboratory and clinical studies of DENV infection and vaccine. Considering the limitations of plaque based assay, some novel methods for neutralizing assays have been described [12-18]. Che and coworkers recently developed a novel ELISPOT based neutralization test, demonstrating a well correlation with the conventional PRNT assay [19]. Pseudo infectious DENV reporter virus particles (RVP) carrying green fluorescent protein (GFP) reporter were also used to measure neutralization antibodies with rapidity, stability and reproducibility [15,16,20]. Infection with RVP could be monitored by the GFP signals using flow cytometry. However, GFP is not suitable for real-time quantification, and production of RVP requires special cell lines and replicon based plasmids. Live reporter virus carrying luciferase reporter replicates almost the same as wild type virus, representing a more advanced tool. Many reporter viruses, including SARS-related corona virus, human hepatitis C virus, parainfluenza virus, HIV, adenovirus, have been described and well applied for antiviral screening, live imaging, or function studies [21-25]. Live reporter DENV engineering a reporter gene at the capsid gene has been developed [26]. Recently, we described the stable luciferase DENV reporter virus Luc-DENV and used it for high-throughput screening for antiviral drugs [9]. In this study, we demonstrated the utility of Luc-DENV for measuring neutralization and enhancing antibodies. Using three identified neutralizing mAbs, Luc-based assay showed well correlation with the PRNT-based assay. 4G2 and 2B8 are both IgG1 isotype mAbs, and 2A10G6 belongs to IgG2a isotype. 2B8 recognizes the domain III of DENV E protein and inhibit viral binding, while 2A10G6 and 4G2 inhibit fusion. All three mAbs were active in inhibiting plaque forming and Luc expression in Luc-DNEV infected Vero cells. The value of PRNT50 and LRNT50 are well correlated (R^2^ > 0.95). The Luc-based assay was readily applied in evaluation of clinical samples from vaccinated animals and infected patients.

ADE infection of DENV has been well demonstrated *in vitro* and *in vivo*, and represents one of the major impediments against vaccine development. Previously, different methods based on infection rate [27,28], progeny viral yield [29], and number of infectious centers [30,31] have been reported to measure the ADE activity in FcR expressing cells including K562, U937 or THP-1 cells. The FACS analysis has been commonly used to quantify the infection rate in C6/36 cells, Raji B, and human peripheral blood mononuclear cells [32,33]. Progeny viral yield can be detected either by conventional plaque assay or NS1-based ELISA [34], ELISPOT [19], and real-time RT-PCR [32]. Recently, Moi *et al.*[35] successfully established stable BHK-21 cell lines that express FcRIIA, which facilitate both neutralization and ADE assay.

The plaque based assay determined the infectious particles released from virus-infected cells, whereas the RLU based assay described in this study offered a simple method which detected viral protein expression in cells. Linear correlation was established between the two assays for both neutralization and ADE assays (Figure 1D and Figure 2B). The newly developed assay method is comparable to the traditional plaque assay, with some unique advantages. First, this Luc-based assay is more substantial and time saving. The conventional plaque test used 12-well plates and 5–7 days observation for the plaque forming, the new test is compared performing the same protocol involved 24-well plates and cost no more than 2 days. Second, this new assay method has a more wide-range scope of application with high repetitiveness and reliability. Luc-DENV replicates well in multiple cells including BHK-21, K562, Vero and THP-1 and A549 cells, and luciferase activity can also be detected stably in various cells. Neutralization and ADE assays can be performed in the same cells [34]. Third, this new assay method is easy to adapt for a high-throughput manner [9], which is of critical importance for large-scale clinical samples assays during clinical trials of dengue vaccine.

## Conclusions

Together, we establish a novel reporter system for neutralizing and enhancing antibody assay against DENV by using an engineered DENV stably expressing *Renilla* luciferase. The newly developed assay described here is rapid, low-cost, and time-saving, providing a useful tool for both basic research and epidemiological investigation.

## Methods

### Cells, virus and antibodies

Baby hamster kidney cells (BHK-21) and African green monkey kidney (Vero) cells were cultured in Dulbecco’s Modified Essential Medium (DMEM; Invitrogen) supplemented with 10% fetal bovine serum (FBS; Hyclone) and 1% penicillin–streptomycin at 37°C in a 5% CO_2_. Human erythroleukemic K562 cells were maintained in RPMI 1640 medium (Invitrogen) supplemented with 10% FBS (GIBCO) at 37°C in a 5% CO_2_. The reporter Luc-DENV has been previously described [9] and was prepared and tittered in Vero cells. The following characterized monoclonal antibodies (mAbs) against DENV were used in this study: 4G2, 2B8 and 2A10G6.

### Clinical samples

Serum samples were collected from Rhesus monkeys (#175, #052) immunized with a single dose of a live attenuated DENV (unpublished data), and serum from the unimmunized animal was set as negative control (#NS). Human convalescent sera from DF patients (#19-20, #37-20, #37-30) and control serum negative for DENV (#NC) were from Guangzhou No.8 People’s Hospital, Guangzhou, China. All samples were inactivated at 56°C for 30 min before assay.

### Plaque reduction neutralization test (PRNT)

PRNT were performed as previously described [12]. Briefly, 2 × 10^5^ cells/well of BHK-21 cells were seeded into 12-well plates and incubated overnight. 100 μl serially diluted antibody samples were mixed with an equal volume of Luc-DENV containing 30 PFU. After 1 h incubation, 200 μL of antibody-virus mixture was added to BHK-21 cell monolayer in 12-well plates for another 1 h. Next, the supernatant was removed, and cells were overlaid with 1 mL of 1.0% (w/v) agarose (Promega) in DMEM containing 4% FBS. After further incubation at 37°C for 4 days, the overlay was removed, and cells were fixed with 4% formaldehyde for 30 min, and stained with 1% (w/v) crystal violet. DMEM served as negative control, and each sample was assayed in triplicate. Plaques were counted and PRNT50 is defined as the antibody dilution resulting in 50% plaque reduction referred to negative control.

### Luc-base neutralization assay

Luc-based neutralization assay was performed in 12-well plates, and the procedure was similar to the conventional PRNT assay. Briefly, virus-antibody mixture was added to BHK-21 cells in 12-well plates and adsorbed for 1 h at 37°C. Supernatant was removed and 1 mL DMEM-2% FBS was replenished onto cells. After 48 h incubation at 37°C, the supernatant was removed, cells were lysed with 250 μl lysates (Promega) per well for 15 minutes. 50 μl lysed suspension was assayed for enzyme activities after adding 100 μl substrate reagent. Data was collected using a continuous-read luminometer (GLOMAX 96 Microplate Luminometer, Promega) integrated over 10 seconds with a 2 second delay. Medium served as negative control, each sample was assay in triplicate. The antibody dilution resulting in 50% reduction of RLU value referred to the negative control was defined as LRNT50.

### Plaque-based enhancement assay

The protocol for ADE assay has been previously described [36]. Briefly, pre-formed antibody-DNEV complex were prepared by incubating serially 10-fold diluted antibody with Luc-DENV at MOI of 0.5 in 37°C before applying to 1 × 10^5^ K562 cells in 12-well plates. Cells were incubated for additional 72 hours, and the virus titer in the supernatant was titrated by standard plaque assay on BHK-21 cells.

### Luc-based enhancement assay

The Luc-based ADE assay was operated similar with plaque-based enhancement assay as above described in 12-well plates. Serial dilutions of antibodies mixed with Luc-DENV were incubated for 72 hours on K562 cells, cell lysates were then subjected to luciferase activities assay as described above. The enhancing activity was evaluated by comparing the RLU value from cells harboring antibody-Luc-DENV complex and that from cells harboring Luc-DENV alone.

### Statistical analysis

All statistical analyses were performed using SPSS 13.0. Graphs were performed using the Prism software (GraphPadPrism5, San Diego, CA). The data were presented as means plus standard deviations from there independent experiments. A *P* value < 0.05 was considered statistically significant.

## Competing interests

The authors declare that they have no competing interests.

## Authors’ contributions

CFQ and KYS conceived and designed the experiments. KYS, HZ, ZYJ, XFL and YQD performed the experiments. KYS and HZ analyzed the data. TJ, SYZ, BZ, EDQ, FCZ and PYS provided reagents and advice. CFQ and KYS wrote the paper. All authors read and approved the final manuscript.

## Supplementary Material

Additional file 1: Figure S1 Growth curve of Luc-DENV on BHK-21 cells expressed by luciferase activity. Cells were infected with virus at MOI of 0.5, collected and lysed at the indicated time points to measure the luciferase activities. Each data point represents the mean obtained in three separate assays with SD (indicated by bars). Click here for file

Additional file 2: Figure S2 Growth curve of Luc-DENV on K562 cells expressed by luciferase activity. Cells were infected with virus at MOI of 0.5, collected and lysed at the indicated time points to measure the luciferase activities. Each data point represents the mean obtained in three separate assays with SDs (indicated by bars). Click here for file
