# Supplementary figures and images for: A novel reporter system for neutralizing and enhancing antibody assay against dengue virus
Source: BMC Microbiol. 2014 Feb 18;14:44. doi: 10.1186/1471-2180-14-44 (PMC3930823; doi:10.1186/1471-2180-14-44)

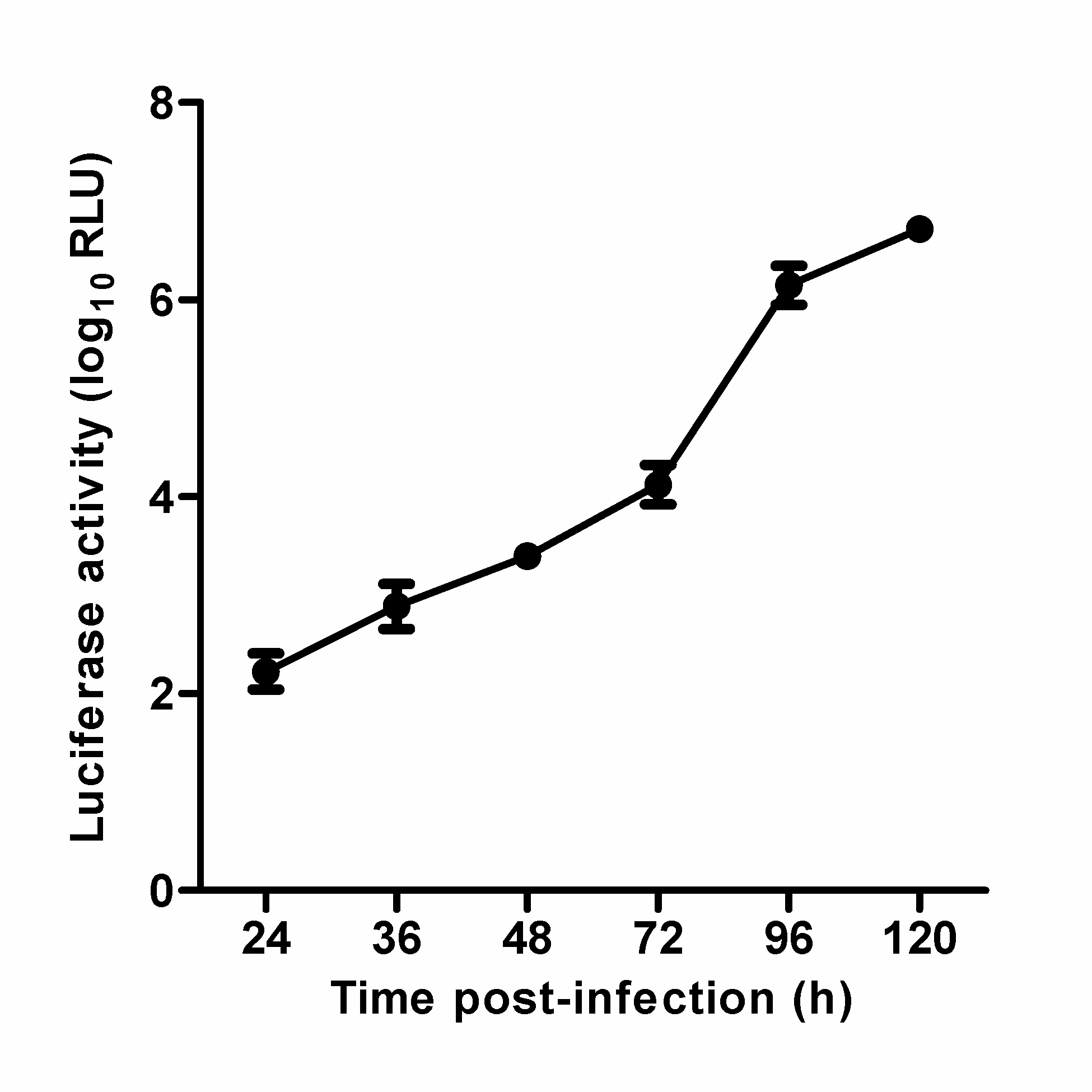

Supplement: Additional file 1: Figure S1 — Growth curve of Luc-DENV on BHK-21 cells expressed by luciferase activity. Cells were infected with virus at MOI of 0.5, collected and lysed at the indicated time points to measure the luciferase activities. Each data point represents the mean obtained in three separate assays with SD (indicated by bars). [file 1471-2180-14-44-S1.tiff]

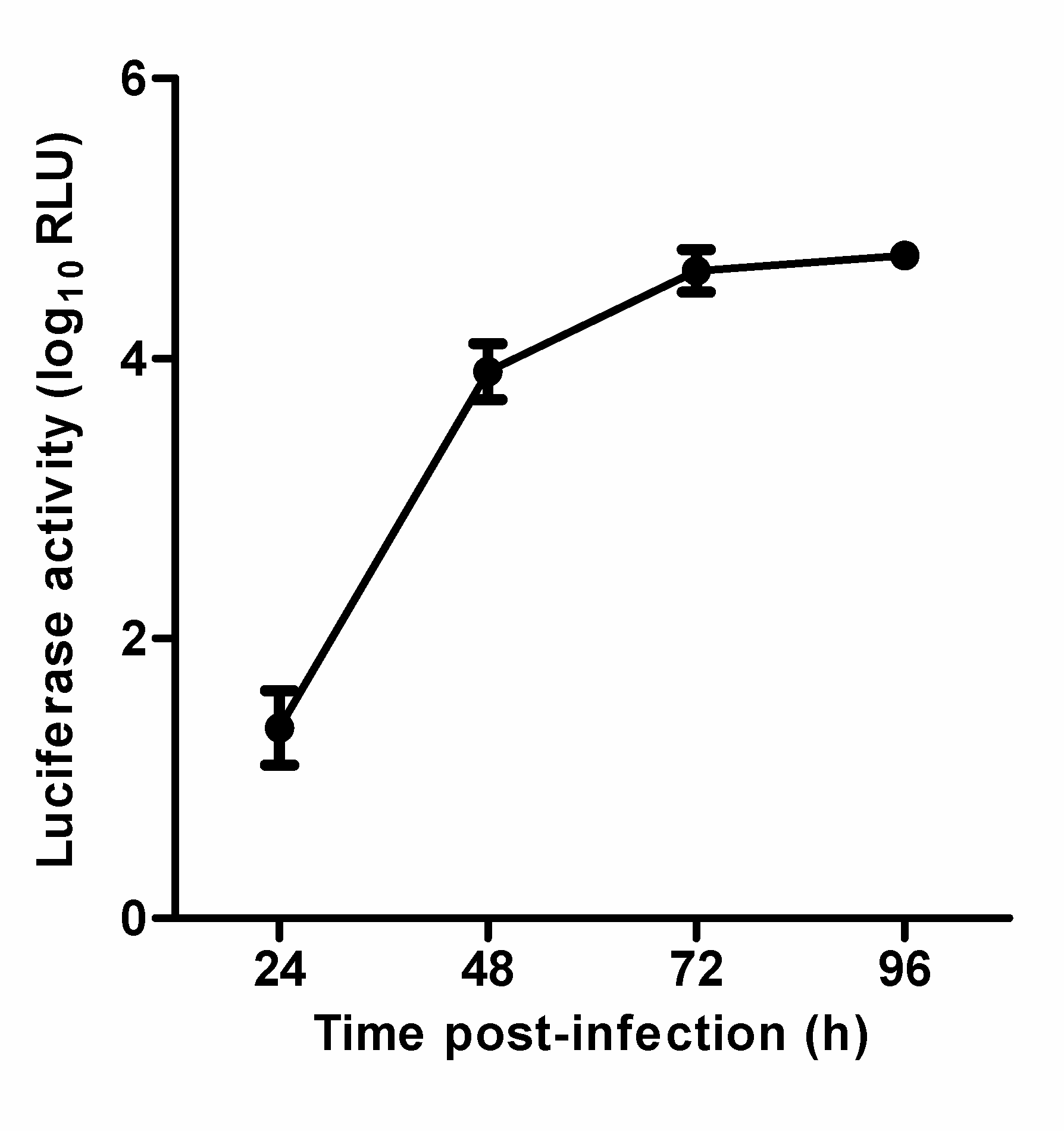

Supplement: Additional file 2: Figure S2 — Growth curve of Luc-DENV on K562 cells expressed by luciferase activity. Cells were infected with virus at MOI of 0.5, collected and lysed at the indicated time points to measure the luciferase activities. Each data point represents the mean obtained in three separate assays with SDs (indicated by bars). [file 1471-2180-14-44-S2.tiff]
